# Development and field validation of a reverse transcription loop-mediated isothermal amplification assay (RT-LAMP) for the rapid detection of chikungunya virus in patient and mosquito samples

**DOI:** 10.1016/j.cmi.2024.03.004

**Published:** 2024-06

**Authors:** Severino Jefferson Ribeiro da Silva, Jurandy Júnior Ferraz de Magalhães, Quinn Matthews, Ana Luisa Lot Divarzak, Renata Pessôa Germano Mendes, Bárbara Nazly Rodrigues Santos, Diego Guerra de Albuquerque Cabral, Jacilane Bezerra da Silva, Alain Kohl, Keith Pardee, Lindomar Pena

**Affiliations:** 1) Laboratory of Virology and Experimental Therapy (Lavite), Department of Virology, Aggeu Magalhães Institute (IAM), Oswaldo Cruz Foundation (Fiocruz), Recife, Pernambuco, Brazil; 2) Leslie Dan Faculty of Pharmacy, University of Toronto, Toronto, ON, Canada; 3) Department of Virology, Pernambuco State Central Laboratory (LACEN/PE), Recife, Pernambuco, Brazil; 4) University of Pernambuco (UPE), Serra Talhada Campus, Serra Talhada, Pernambuco, Brazil; 5) Public Health Laboratory of the XI Regional Health, Pernambuco, Brazil; 6) MRC-University of Glasgow Centre for Virus Research, Glasgow, UK; 7) Department of Vector Biology and Tropical Disease Biology, Liverpool School of Tropical Medicine, Liverpool, UK; 8) Department of Mechanical and Industrial Engineering, University of Toronto, Toronto, ON, Canada

**Keywords:** CHIKV, Diagnostic, Mosquitoes, Patients, Point-of-care, RT-LAMP

## Abstract

**Objectives:**

We aimed to develop a reverse transcription loop-mediated isothermal amplification (RT-LAMP) platform for the rapid detection of chikungunya virus (CHIKV) in both patient and mosquito samples from Brazil.

**Methods:**

We optimized an RT-LAMP assay and then evaluated the specificity and sensitivity using visual detection. In comparison with the RT-qPCR reference method, we validated the utility of this assay as a molecular diagnostic test in a reference laboratory for arbovirus diagnostics using 100 serum samples collected from suspected CHIKV cases.

**Results:**

Our RT-LAMP assay specifically detected CHIKV without cross-reactivity against other arboviruses. The limit of detection of our RT-LAMP was estimated in −1.18 PFU (confidence interval [CI] ranging from –2.08 to 0.45), resulting in a similar analytical sensitivity when directly compared with the reference standard RT-qPCR assay. Then, we demonstrate the ability of our RT-LAMP assay to detect the virus in different human specimens (serum, urine, and saliva), and crude lysate of *Aedes aegypti* mosquitoes in as little as 20–30 minutes and without a separate RNA isolation step. Lastly, we showed that our RT-LAMP assay could be lyophilized and reactivated by adding water, indicating potential for room-temperature storage. Our RT-LAMP had a clinical sensitivity of 100% (95% CI, 90.97–100.00%), clinical specificity of 96.72% (95% CI, 88.65–99.60%), and overall accuracy of 98.00% (95% CI, 92.96–99.76%).

**Discussion:**

Taken together, these findings indicate that the RT-LAMP assay reported here solves important practical drawbacks to the deployment of molecular diagnostics in the field and can be used to improve testing capacity, particularly in low- and middle-income countries.

## Graphical abstract

**Figure:**
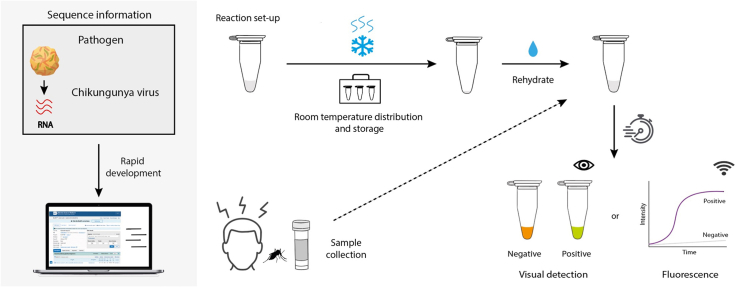

## Introduction

Chikungunya virus (CHIKV) is an arbovirus member of the genus *Alphavirus*, family *Togaviridae* [1]. In most patients, CHIKV infection is typically accompanied by a sudden onset of fever, myalgia, vomiting, rash, nausea, fatigue, headache, and arthralgia, with severe cases causing neurological manifestations observed in the elderly [2,3] and/or chronic polyarthralgia that can persist for months [4,5]. In countries affected by other endemic arboviruses, such as Zika virus (ZIKV) and dengue virus (DENV), the clinical diagnosis of CHIKV infection becomes extremely difficult because of the similar clinical presentation.

Currently, RT-qPCR is considered the reference method for the detection of CHIKV from patient and mosquito samples [6]. Although sensitive and specific, RT-qPCR requires specialized laboratory instrument and involves the use of multistep protocols, leading to limited availability outside of high-resource settings and slow turnaround time of several days or weeks, thus delaying clinical decision-making and therapeutic management of patients.

An alternative to RT-qPCR is reverse transcription loop-mediated isothermal amplification (RT-LAMP) [[7], [8], [9], [10], [11]], which has been widely used for the detection of RNA viruses such as ZIKV [7], DENV [12], Ebola virus [13], and SARS-CoV-2 [[14], [15], [16]]. This isothermal amplification method enables rapid and sensitive detection of a biomarker gene sequences [10] and, unlike RT-qPCR, RT-LAMP amplification of viral target occurs at a constant temperature and the results can be easily read with the naked eye [7,8,17]. Thanks to its notable simplicity and high performance, much effort has been put toward the development of RT-LAMP assays during the COVID-19 pandemic, and the potential of field application has been recognized [[17], [18], [19], [20]].

Here, we report a rapid, low-cost RT-LAMP assay for the detection of CHIKV in different human samples, including serum, saliva, urine, and crude lysate of *Aedes aegypti* mosquitoes*.* We then demonstrate the ability to lyophilize our RT-LAMP platform, indicating potential for room-temperature storage.

## Methods

### Synthetic RNA control for molecular reactions

DNA encoding the CHIKV target was obtained from the Sangon Biotech Co. (China) in a pUC57 backbone. *In vitro* T7 RNAP-based transcription of synthetic RNA was done overnight using the HiScribe T7 Quick High Yield RNA Synthesis Kit (E2050S, NEB) following the manufacturer's instructions.

### Cells and viruses

Vero cells were maintained in Dulbecco's modified Eagle's medium (Gibco, Carlsbad, CA) at 37°C under 5% CO_2_. CHIKV strain PE2016-480 used in this study belongs to the East/Central/South African (ECSA) genotype (unpublished) and was isolated from a patient serum in Pernambuco State, Brazil. Similarly, other arboviruses (DENV [[1], [2], [3], [4]], YFV [yellow fever virus], ZIKV, and MAYV [Mayaro virus]) were propagated in Vero cells and titrated by plaque assay.

### Optimization of the CHIKV RT-LAMP assay

To optimize RT-LAMP for CHIKV detection, different reaction settings were tested as we have previously described for ZIKV [9]. Briefly, 25 μL RT-LAMP reactions were prepared in triplicates containing 1 × buffer, 4 U Bst DNA Polymerase [version 3.0, NEB], 8 mM MgSO_4_, 1.4 mM deoxynucleotides triphosphates (dNTPs) (ThermoFisher Scientific), 0.2 μM for F3, 0.2 μM for B3, 1.6 μM for FIP, 1.6 μM for BIP, 0.4 μM for LF, 0,4 μM for LB primers, and 5.0 μL of template. The WarmStart LAMP 4X Master Mix (Lyo-compatible, M1710B, NEB) were assembled in 25 μL following the manufacturer's instructions. The CHIKV RT-LAMP primers targeted the envelope gene (6K-E1) of the genome in both genotypes (Asian and ECSA) (Supplementary Data 1) [21]. All reactions were set-up on ice, incubated at 65°C for 30 minutes, then inactivated at 80°C for 5 minutes. RT-LAMP amplicons were analyzed using four different approaches as described previously [9]. For real-time detection, amplicons were visualized by adding 0.5 μL of 1 × LAMP Fluorescent Dye (B1700S, NEB).

### Lyophilization of RT-LAMP reactions and storage at room temperature

Unless otherwise mentioned, RT-LAMP reactions were mixed as described above (excluding dye and sample) and aliquoted into 1.5 mL tubes. The tubes were then punctured twice in the cap with a 22 G needle and frozen using liquid nitrogen. Tubes were then rapidly transferred into a vacuum concentrator connected to the lyophilizer equipment (FreeZone 6 L −84°C Console Freeze Dryer) and lyophilized as described previously [22].

### Viral RNA extraction and RT-qPCR assay for CHIKV detection

Viral RNA was extracted from samples using QIAamp Viral RNA Mini Kit (52906, Qiagen) according to the manufacturer's instructions. Samples were assayed for positivity and analyzed by RT-qPCR according to protocols established by the US CDC (positive samples: cycle quantification [Cq] ≤37) [6]. Primers and probe were synthetized by Integrated DNA Technologies (IDT) and can be found in Supplementary Data 1. The RNA copy numbers of CHIKV in each reaction was estimated by comparing the Cq values to the standard curve made by ten-fold serial dilutions of CHIKV transcript (Fig. S1).

### Analytical specificity and sensitivity of RT-LAMP assay for CHIKV detection

To determine the analytical specificity of the CHIKV RT-LAMP, the primers were tested to detect only CHIKV against a panel of different arboviruses (Table S1). To evaluate the analytical sensitivity of the RT-LAMP assay, a ten-fold serial dilution of CHIKV was made in human serum. Virus concentration in spiked specimens ranged from 10^5^ to 10^−7^ PFU. In parallel, viral RNA from the same dilutions was extracted and then tested with the RT-qPCR method [6].

### Human biological and mosquito samples experiments under controlled conditions

To evaluate the ability of the RT-LAMP to detect CHIKV in human and mosquito samples, we spiked serum, saliva, urine, and crude lysate of *Ae. Aegypti* mosquitoes with two clinically viral loads: 10^6^ PFU/mL and 10^3^ PFU/mL, as described previously [9].

### Field validation using patient samples

A total of 100 clinical samples were obtained from patients in Pernambuco State, Brazil, with suspected arbovirus infection during the triple epidemic of ZIKV, DENV, and CHIKV between 2016 and 2018. Briefly, venous whole blood samples were collected in EDTA tubes and separated plasma/sera samples were kept at –80°C until use. Viral RNA was extracted as described above.

### Sequencing of the CHIKV RT-LAMP amplicons

Genetic characterization of the RT-LAMP amplicons and DNA analysis from one patient sample was performed by the Sanger method as described previously [9].

### Statistics

Graphs and analysis were done using GraphPad Prism (version 10, GraphPad Software) and MedCalc software (version 19.2.0, MedCalc Software, Ostend, Belgium). Diagnostic parameters were calculated using an online mathematical tool provided by MedCalc (https://www.medcalc.org/calc/diagnostic_test.php).

### Ethics

This study was approved by the Pernambuco State Haematology and Hemotherapy Foundation (HEMOPE-PE), Institutional Review Board (IRB) under protocol CAAE: 43877521.4.00000.5195. Informed consent of all patients included in the present study was waived by the IRB for diagnostic samples.

## Results

### Establishing an RT-LAMP assay for CHIKV detection

To develop an affordable RT-LAMP assay for use in limited-resource settings, we first explored different RT-LAMP mix formulations: the WarmStart LAMP Master Mix and an RT-LAMP formulation containing Bst DNA polymerase 3.0. Side-by-side testing of these mixes demonstrated similar performance in terms of colorimetric readout and real-time detection (Figs S2(a) and (b)). Despite its efficiency, the high cost of WarmStart LAMP Master Mix could limit its use in low-resource settings. Comparatively, we previously developed an RT-LAMP assay containing Bst DNA polymerase 3.0 at a cost per reaction of <USD 1 [9], a stark contrast to the USD ∼4 per reaction for WarmStart LAMP Mix. Given the versatility and low cost, we opted to use an RT-LAMP formulation containing Bst DNA polymerase 3.0 and the colorimetric readout for the next phase of the project.

### Establishing the optimal protocol for visual detection

Optimization is a critical step in the development of an RT-LAMP assay. We began by screening incubation time, temperature, concentrations of magnesium, dNTPs, dye (SYBR Green I), and primer combinations. We found that CHIKV genome amplification (10^5^ PFU, cultured virus) occurred at 30 minutes after incubation (Fig. S3(a)). Positive amplification was detected at incubation temperatures ranging from 59°C to 68°C (Fig. S3(b)). Optimal reagents concentrations were identified as 8 mM Mg^2+^ and 1.4 mM dNTPs, and SYBR Green I was found to perform optimally at a 1:10 dilution (Figs S3(c), (d), and (f)). All three sets of LAMP primers were required for amplification of the CHIKV genome (Fig. S3(e)). These optimal parameters were selected and used for further experiments, as described below.

### Analytical specificity of CHIKV RT-LAMP assay

We next determined the analytical specificity of the assay using a panel of several endemic arboviruses in Latin America that could, in practice, be found in individuals presenting clinical symptoms typically attributed to CHIKV infection under real-life settings. Positive results were obtained only in samples containing CHIKV (Fig. S4). Parallel testing using RT-qPCR agreed with these findings, in which the Cq value for the CHIKV sample was 12.7.

### Analytical sensitivity of CHIKV RT-LAMP assay

Our RT-LAMP assay could detect a broad range of CHIKV concentration (from 10^5^ to 10^−5^ PFU) (Fig. S5). In parallel, these CHIKV serum dilutions were extracted and tested by RT-qPCR. The analytical sensitivity of RT-qPCR was only observed down to 10^−1^ PFU with a Cq value of 33.3 (Figs S5 and S6). Probit regression analysis was used to accurately determine the limit of detection of our RT-LAMP assay using 10 replicates of each dilution. The limit of detection of RT-LAMP at 95% probability was estimated in −1.18 PFU (confidence interval [CI] ranging from –2.08 to 0.45), indicating that our RT-LAMP assay exhibits an equivalent analytical sensitivity to the reference standard RT-qPCR assay (Fig. S7 and Table S2).

### Detection of CHIKV in virus-spiked human and mosquito samples

**Fig. 1:**
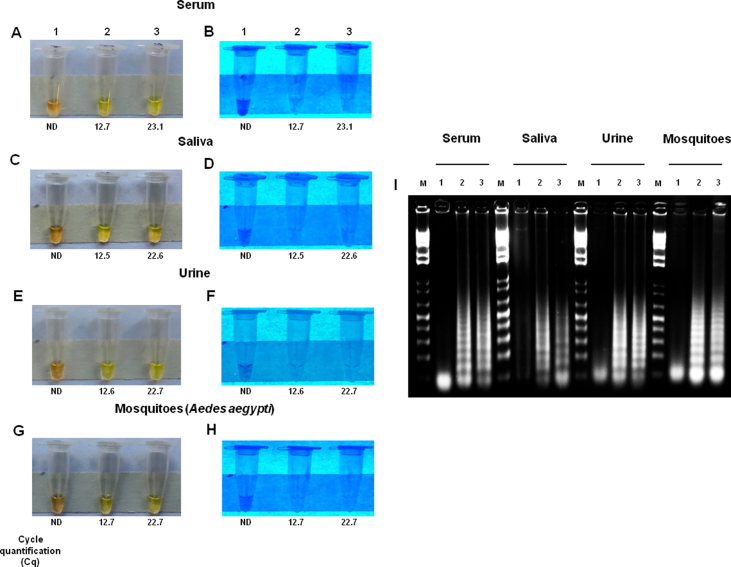
CHIKV detection in virus-spiked specimens. Specimens including serum, saliva, urine and crude lysate of *Aedes aegypti* mosquitoes were spiked with two viral loads: 10^6^ or 10^3^ PFU/mL. Then, these samples were directly assayed (a–i). To compare the results of RT-LAMP with RT-qPCR, viral RNA was extracted from the same specimens and then tested. The Cq values are described at the bottom of the figure. RT-LAMP products were visualized by three different methods: naked eye as visualized by addition of SYBR Green I (a, c, e, g), fluorescence under UV light (b, d, f, h), or looking for a typical band pattern of a successful RT-LAMP reaction using gel electrophoresis (2 %) (i). Legends in (a–i) are (1) uninfected biological specimen (serum, saliva, urine, and crude lysate of *Ae. aegypti* mosquitos); (2) specimen spiked with 10^6^ PFU/mL; (3) specimen spiked with 10^3^ PFU/mL. CHIKV, Chikungunya virus; Cq, cycle quantification; M: molecular weight marker; ND, not detected; RT-LAMP, reverse transcription loop-mediated isothermal amplification.

We next evaluated the ability of our RT-LAMP assay to detect CHIKV in human biofluids and mosquito samples under controlled conditions. Our RT-LAMP assay was able to detect CHIKV in all spiked samples in both clinically viral loads (Fig. 1(a)–(i)). All specimens were assayed in parallel using RT-qPCR, reporting Cq values (12.7; 12.5; 12.6; and 12.7) and (23.1; 22.6; 22.7; and 22.7), for both viral loads in serum, saliva, urine, and crude lysate of *Ae. aegypti*, respectively (Fig. S8).

### Clinical validation in a reference laboratory for arbovirus diagnostics in Brazil

**Fig. 2:**
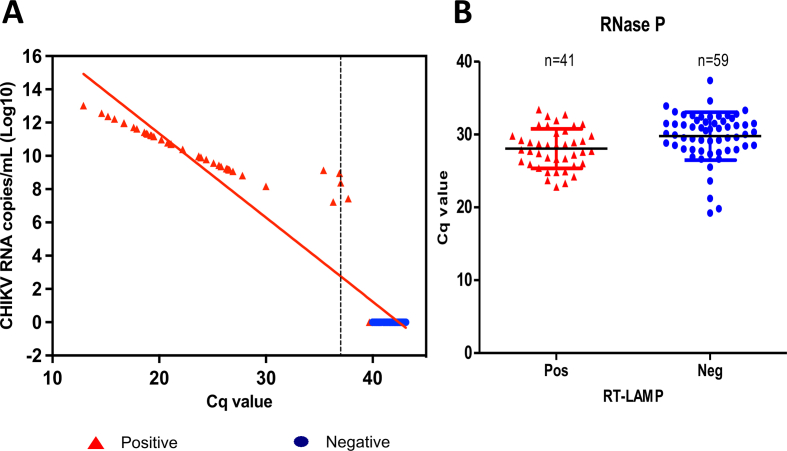
Performance of the diagnostic system in a reference laboratory. A total of 100 serum samples were tested for CHIKV by RT-LAMP and RT-qPCR. Of these, 39 were positive and 61 were negative for CHIKV as determined by RT-qPCR and 41 were positive and 59 were negative for CHIKV as determined by RT-LAMP (a). The same samples were tested using the RT-qPCR protocol for detection of the endogenous Ribonuclease P (RNAse P) gene, where the Cq value in these specimens ranged from 19.2 to 37.4 (b). According to the RT-qPCR protocol used, the Cq threshold for CHIKV positivity was ≤37 (represented by the dashed line in panel a) and the Cq threshold for RNase P endogenous control was ≤40. Red triangles represent specimens identified as positive and blue circles represent specimens identified as negative by our RT-LAMP assay. CHIKV, Chikungunya virus; Cq, cycle quantification; RT-LAMP, reverse transcription loop-mediated isothermal amplification.

We began field validation using patient samples collected from suspected arbovirus infection in Brazil. As the site for the trial, we selected the reference laboratory for arboviruses diagnostics at Fiocruz Pernambuco, Recife, Pernambuco State, which is an area known for simultaneous circulation of arboviruses [23,24]. A total of 100 serum specimens double blinded (61 negative and 39 positive samples for CHIKV, as determined by RT-qPCR) were used with Cq values ranging from 12.9 to >40.0. Our RT-LAMP assay detected CHIKV in 41 specimens, including two specimens that had been deemed negative by RT-qPCR. In contrast, 59 were determined as negative by RT-LAMP (Fig. 2(a)). Integrity of RNA in the samples was confirmed using RT-qPCR for RNase (Fig. 2(b)). Furthermore, specimens at the detection threshold by RT-qPCR (with Cq values; 35.4, 36.3, 36.9, 37.0, and 37.7) were assayed by RT-LAMP and all reactions showed a positive result, including one sample that had been deemed negative by RT-qPCR with Cq value 37.7 (Fig. S9). Using the Sanger method, we sequenced the second RT-LAMP positive sample that had been deemed indeterminate by RT-qPCR, confirming the accuracy of our test to detect the virus (Fig. S10).

In comparison with the RT-qPCR technique, the CHIKV RT-LAMP assay had a clinical sensitivity of 100% (95% CI, 90.97–100.00%) and clinical specificity of 96.72% (95% CI, 88.65–99.60%). The overall accuracy of the CHIKV RT-LAMP assay was 98.00% (95% CI, 92.96–99.76%) (Table 1). Taken together, these findings indicate that our RT-LAMP assay exhibits diagnostic performance equivalent to RT-qPCR, highlighting the potential for our assay to be used in clinical settings.

**Table 1:** Diagnostic performance of RT-LAMP assay for CHIKV diagnostics using patient samples

	RT-qPCR +	RT-qPCR –	Total
RT-LAMP +	39	2	41
RT-LAMP –	0	59	59
Total	39	61	
Sensitivity	100 % (95% CI, 90.97–100.00%)		
Specificity	96.72 % (95% CI, 88.65–99.60%)		
CHIKV prevalence	39.00% (95% CI, 29.40–49.27%)		
Positive Predictive Value (PPV)	95.12% (95% CI, 83.30–98.70%)		
Negative Predictive Value (NPV)	100 % (95% CI, 93.94–100.00%)		
Accuracy	98.00% (95% CI, 92.96–99.76%)		

CHIKV, Chikungunya virus; RT-LAMP, reverse transcription loop-mediated isothermal amplification.

### Establishing a lyophilized RT-LAMP assay for CHIKV detection

Field-deployable diagnostics are urgently needed to combat infectious diseases. With this perspective in mind, we have established a lyophilized RT-LAMP for CHIKV diagnostics (Figs S11(a) and (b)). This started with evaluation of lyophilized and fresh reactions using *in vitro*-transcribed RNA inputs (300 nM), indicating that our RT-LAMP could be lyophilized and reactivated by adding water (Fig. S11(c)). Lastly, we demonstrate that our RT-LAMP platform could be lyophilized and stored at room temperature for ≥2 weeks (Figs S11(d) and (e)), suggesting potential for room-temperature storage and distribution without cold chain logistics.

## Discussion

Given the lack of readily available vaccines against CHIKV infection, timely and reliable CHIKV diagnosis remains the primary defence against disease spread. In remote and low-resource areas, field-deployable point-of-care (PoC) diagnostics are needed to fill this role, ensuring that patients receive timely treatment. Building upon our previous development of PoC diagnostics for Ebola virus [25], ZIKV [8,9,[26], [27], [28]], and SARS-CoV-2 [29], here we present a collaborative effort to develop an RT-LAMP assay for detection of CHIKV.

To facilitate the use of our RT-LAMP assay in real-life settings and further prevent false-positives, we identified minimum sample/reaction handling/processing as a critical requirement. Typically, RT-LAMP is performed using a two-step protocol in which two enzymes—a reverse transcriptase and a DNA polymerase—are required, as well as a preceding RNA isolation step. To simplify this, we opted to use Bst DNA polymerase 3.0 (NEB), a single enzyme that exhibits both reverse transcriptase and polymerase activity [7], resulting in a one-step protocol. Although several two-step RT-LAMP protocols have been described for CHIKV detection [21,30], the one-step protocol described here overcomes the need for multiple enzymes and sample processing steps.

A key consideration when evaluating PoC diagnostics is the ability to detect viral RNA in patients with low viral load. Serum samples collected from patients 1–8 days after illness onset typically have a high viral load (1 × 10^5^ copies/mL) [31]. In contrast, a significant reduction in CHIKV viral load (1 × 10^2^–1 × 10^1^ copies/mL) is usually observed in samples collected 8 days after illness onset [31]. Here, we showed that our RT-LAMP assay was able to provide detection well within clinically relevant concentrations (from 10^5^ to 10^−5^ PFU)—a similar analytical sensitivity to RT-qPCR. In agreement with our findings, some reports have documented that the analytical sensitivity of RT-LAMP is similar or even superior to RT-qPCR for CHIKV detection [21,30,32].

Several other platforms have been developed for CHIKV diagnostics. However, PoC deployment of many of these technologies is dependent on multiple liquid-handling steps, reliable access to electricity, technically skilled operators, and sophisticated and proprietary hardware [21,[32], [33], [34], [35]]. Moreover, many of these technologies have only been evaluated using spiked-virus samples or with a limited number of patient samples.

Our RT-LAMP assay comes at a significantly reduced cost when compared with RT-qPCR and RT-LAMP commercial kits. The cost of each RT-LAMP reaction in this study was <USD 1, a meaningful contrast to the USD 11–25 required for each RT-qPCR test [9,15] and USD 4 required for RT-LAMP reactions using commercial kits. However, we believe that this cost can be reduced even further by the potential replacement of commercial research-grade enzymes with locally manufactured reagents [36,37].

In summary, we have developed a rapid, low cost, lyophilized RT-LAMP assay for the detection of CHIKV in patient and mosquito samples. Overall, our RT-LAMP assay displayed high accuracy and is an inexpensive molecular platform for the diagnosis of CHIKV, and may serve as a basis for the development of alternative diagnostic methods that can be used for PoC applications.

## Author contributions

SJRS and LP conceived the work. SJRS, JJFM, ALLD, DGAC, and JBS conducted the experiments. SJRS and LP performed data analysis and interpretation. SJRS wrote the original draft. SS, QM, RPGM, BNRS, AK, KP, and LP wrote the final draft. SS and LP supervised the work. All authors critically revised and approved the final version of the manuscript.

## Transparency declaration

SJRS and LP are the inventors of the RT-LAMP protocol described throughout this study and both authors have other patents related to this technology (BR 10 2019 027711 4, filed 23 December 2019). The other authors declare that they have no conflicts of interest. This work was supported by funds to LP and KP from the International Development Research Centre (IDRC, Grant No. 109434). LP is funded by the Fiocruz Inova Programme, Canadian International Development Research Centre (108410-001) and the Foundation for Science and Technology of Pernambuco – FACEPE, Brazil (Grant No. APQ-0154-2.12/16). SJRS was supported by a Ph.D. fellowship sponsored by the Foundation for Science and Technology of Pernambuco (FACEPE), reference number IBPG-1321-2.12/18, and currently is supported by a Postdoctoral Fellowship sponsored by the University of Toronto, Canada. JJFM is funded by the Foundation for Science and Technology of Pernambuco – FACEPE, Brazil (Grant No. APQ-0741-4.01/22). AK is funded by the UK Medical Research Council (MC_UU_12014/8, MC_UU_00034/4). The funders had no role in study design, data collection and analysis, decision to publish, or preparation of the manuscript.
