# Supplementary material for: Development and field validation of a reverse transcription loop-mediated isothermal amplification assay (RT-LAMP) for the rapid detection of chikungunya virus in patient and mosquito samples
Source: Clin Microbiol Infect. 2024 Jun;30(6):810–5. doi: 10.1016/j.cmi.2024.03.004 (PMC11161457; doi:10.1016/j.cmi.2024.03.004)
Supplement: Multimedia component 2 [file mmc2.docx]

Supplementary information for:

**Development and field validation of an RT-LAMP assay for the rapid detection of chikungunya virus in patient and mosquito samples**

Severino Jefferson Ribeiro da Silva^1,2*^, Jurandy Júnior Ferraz de Magalhães^1,3,4,5^, Quinn Matthews^2^, Ana Luisa Lot Divarzak^2^, Renata Pessôa Germano Mendes^1^, Bárbara Nazly Rodrigues Santos^1^, Diego Guerra de Albuquerque Cabral^3^, Jacilane Bezerra da Silva^3^, Alain Kohl^6,7^, Keith Pardee^2,8^, Lindomar Pena^1*^

^1^Laboratory of Virology and Experimental Therapy (Lavite), Department of Virology, Aggeu Magalhães Institute (IAM), Oswaldo Cruz Foundation (Fiocruz), 50670-420, Recife, Pernambuco, Brazil.

^2^Leslie Dan Faculty of Pharmacy, University of Toronto, Toronto, ON M5S 3M2, Canada.

^3^Department of Virology, Pernambuco State Central Laboratory (LACEN/PE), Recife, Pernambuco, Brazil.

^4^University of Pernambuco (UPE), Serra Talhada Campus, 56909-335 Serra Talhada, Pernambuco, Brazil.

^5^Public Health Laboratory of the XI Regional Health, 56912-160 Serra Talhada, Pernambuco, Brazil

^6^MRC-University of Glasgow Centre for Virus Research, Glasgow G61 1QH, UK.

^7^Department of Vector Biology and Tropical Disease Biology, Liverpool School of Tropical Medicine, Liverpool L3 5QA, UK.

^8^Department of Mechanical and Industrial Engineering, University of Toronto, Toronto, ON, Canada.

*Co-corresponding authors:

Severino Jefferson Ribeiro da Silva, Ph.D. Emails: [jeffersonbiotecviro@gmail.com](mailto:jeffersonbiotecviro@gmail.com) , [jefferson.silva@utoronto.ca](mailto:jefferson.silva@utoronto.ca)

Lindomar Pena, Ph.D. Email: [lindomar.pena@fiocruz.br](mailto:lindomar.pena@fiocruz.br)

**
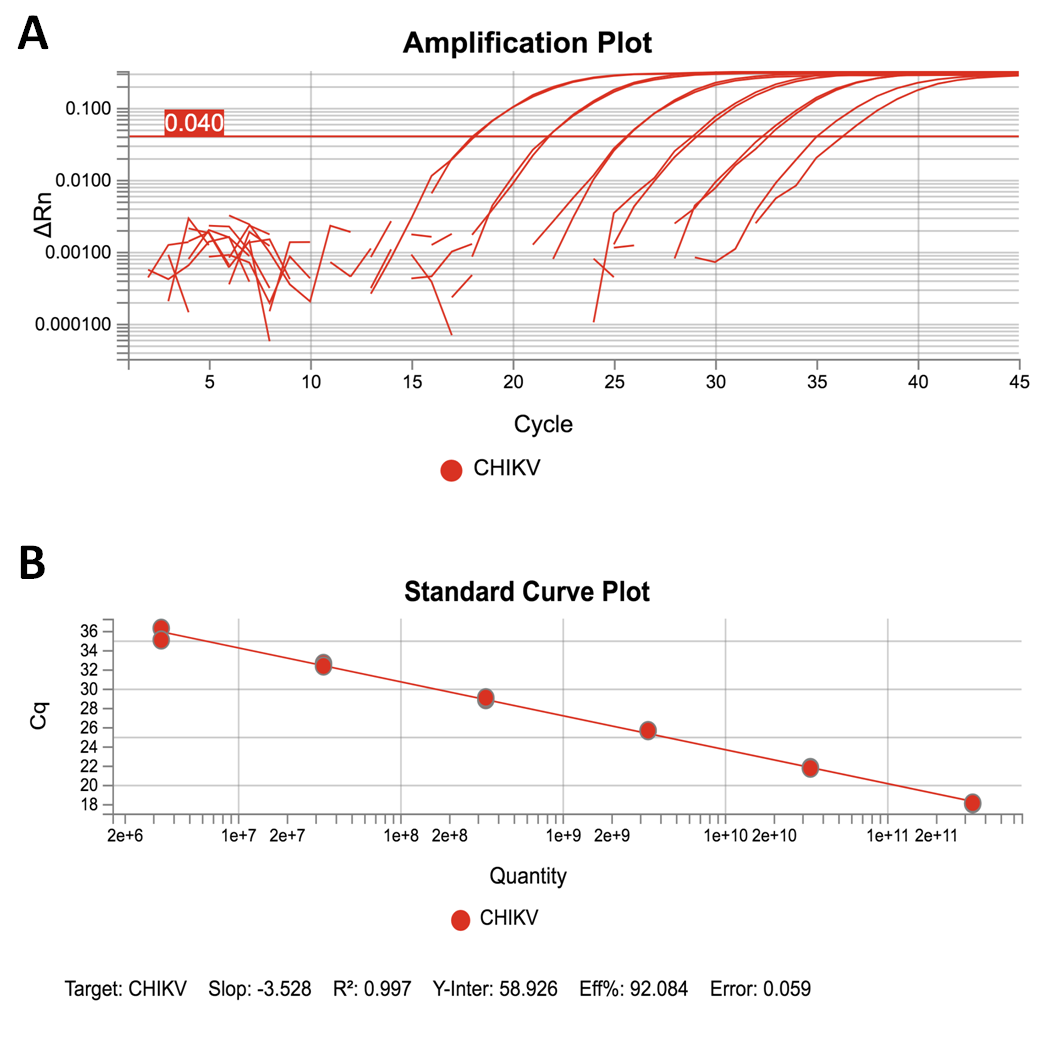
**

**Figure S1. Amplification and standard curve plots obtained from the RT-qPCR used during the field validation using patient samples.**

**
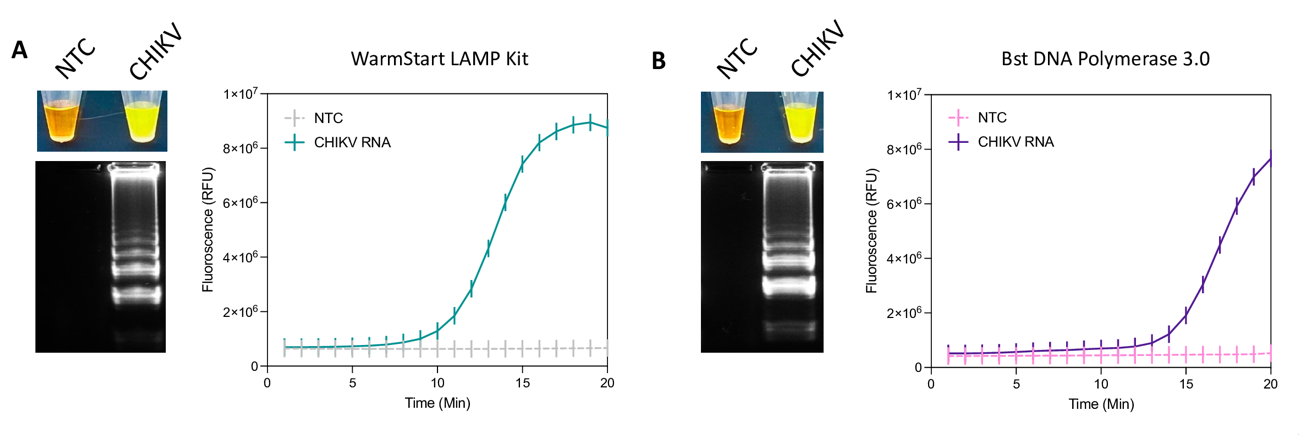
Figure S2. Evaluation of two different RT-LAMP mix formulations.** Colorimetric and real-time detection of CHIKV using WarmStart LAMP Master Mix® (A). Colorimetric and real-time detection of CHIKV using an RT-LAMP mix containing Bst DNA polymerase 3.0 (B). Legends: NTC: non-template control; RFU: relative fluorescence unit.


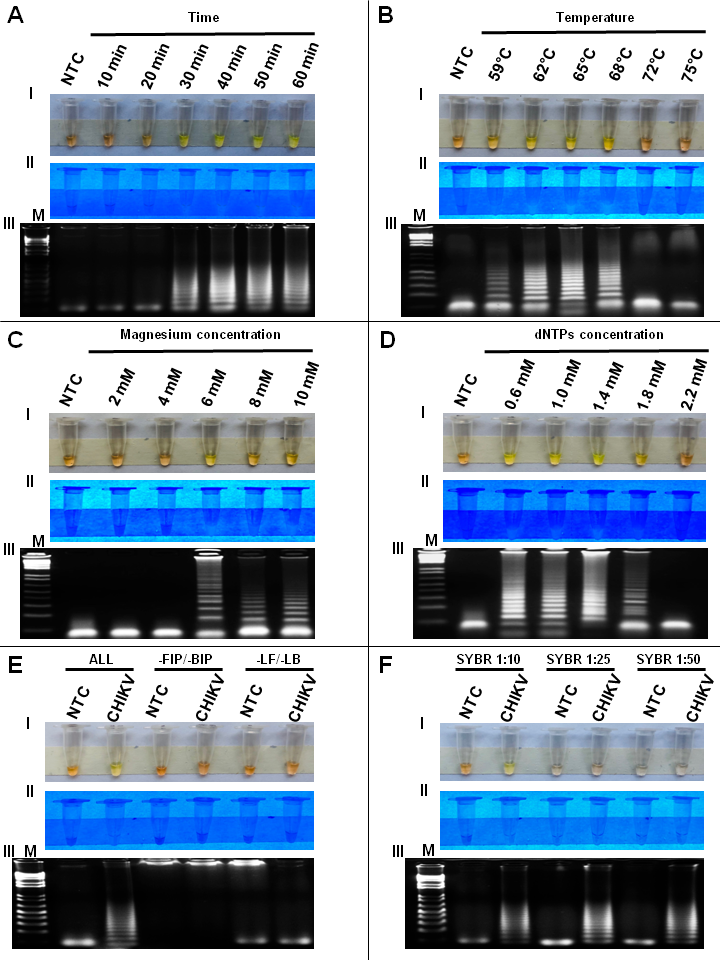
**Figure S3. Optimization of reagent concentrations and conditions of the CHIKV RT-LAMP assay.** All reagent concentrations and reactions settings were first optimized. RT-LAMP reactions were conducted under incubation times ranging from 10 to 60 min (A) and temperatures ranging from 59 to 75 °C (B). Optimal concentrations of magnesium (ranging from 2 to 10 mM) (C) and dNTPs (ranging from 0.6 to 2.2 mM) (D) were determined. Testing of primers sets indicated that all primers are required to perform the RT-LAMP reaction. Reactions without internal (FIP and BIP) or loop (LF and LB) primers resulted in a negative RT-LAMP reaction (E). SYBR Green dye concentrations (1:10, 1:25 and 1:50) were also evaluated to define the optimal concentration to be used in the visual interpretation of results by naked eye (F). RT-LAMP products were visualized by three different methods: naked eye as visualized by addition of SYBR Green I (top panel), fluorescence under UV light (middle panel), or looking for a typical band pattern of a successful RT-LAMP reaction using gel electrophoresis (2 %) (bottom panel). Legends: NTC: non-template control; M: molecular weight marker.

**
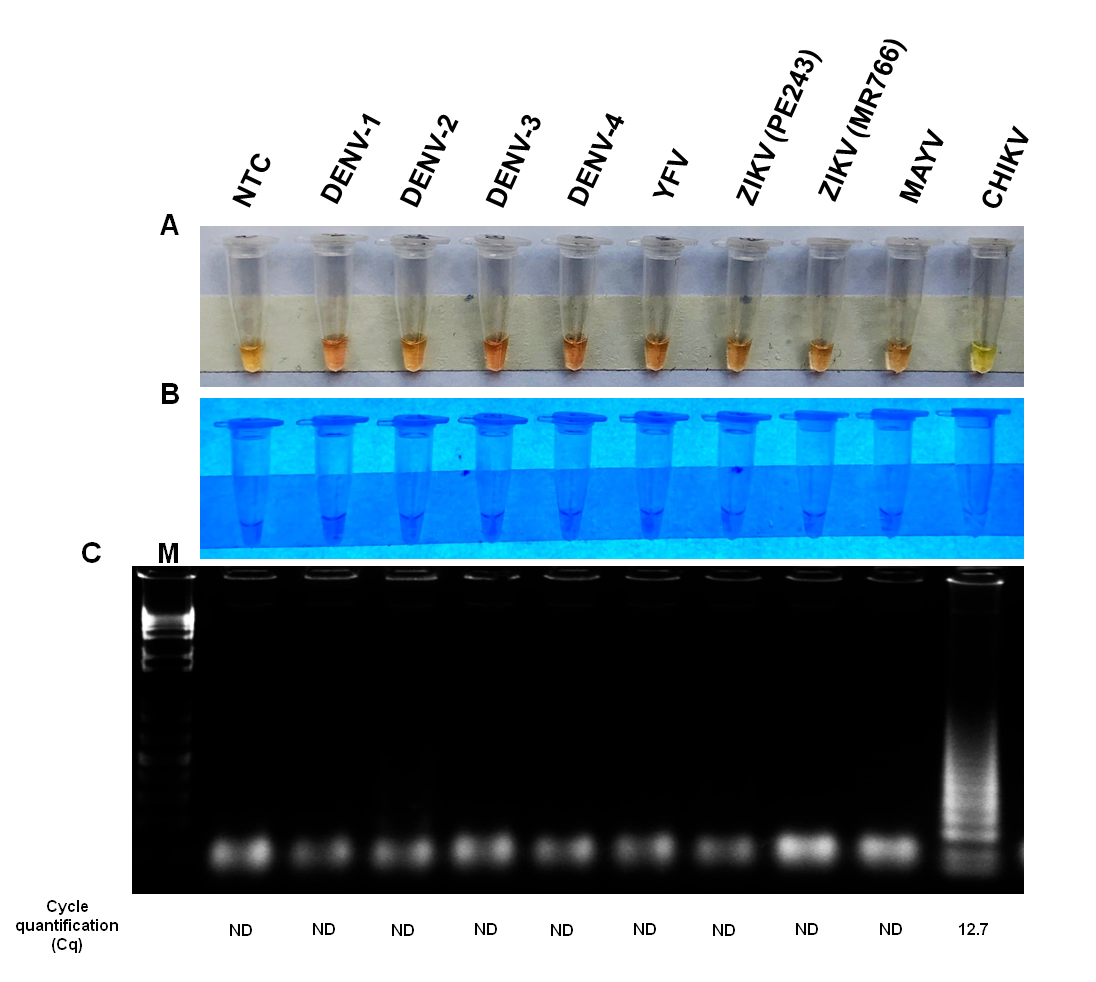
**

**Figure S4. Analytical specificity of CHIKV RT-LAMP.** Specificity assessment of RT-LAMP was evaluated by spiking different arboviruses into uninfected human serum. To compare RT-LAMP results with the RT-qPCR method, the samples underwent RNA extraction and were tested. The Cq values are described at the bottom of the figure. RT-LAMP products were visualized by three different methods: naked eye as visualized by addition of SYBR Green I (top panel), fluorescence under UV light (middle panel), or looking for a typical band pattern of a successful RT-LAMP reaction using gel electrophoresis (2 %) (bottom panel). Legends: NTC: non-template control; M: molecular weight marker; ND: not detected.

**
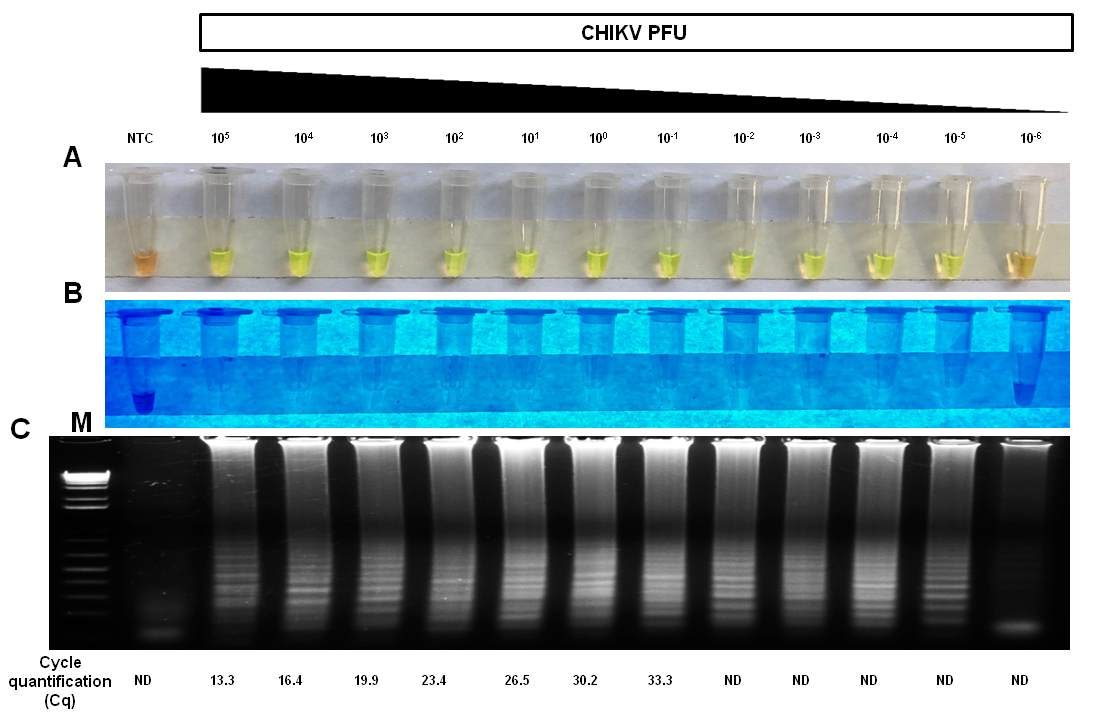
**

**Figure S5. Analytical sensitivity of CHIKV RT-LAMP.** Sensitivity assessment of RT-LAMP was determined by spiking different amounts (10^5^ to 10^–6^ PFU) of CHIKV into uninfected human serum. To compare the results of RT-LAMP with RT-qPCR, viral RNA was extracted from the same dilutions and then tested. The Cq values are described at the bottom of the figure. RT-LAMP products were visualized by three different methods: naked eye as visualized by addition of SYBR Green I (top panel), fluorescence under UV light (middle panel), or looking for a typical band pattern of a successful RT-LAMP reaction using gel electrophoresis (2 %) (bottom panel). Legends: M: molecular weight marker; NTC: non-template control; ND: not detected.

**
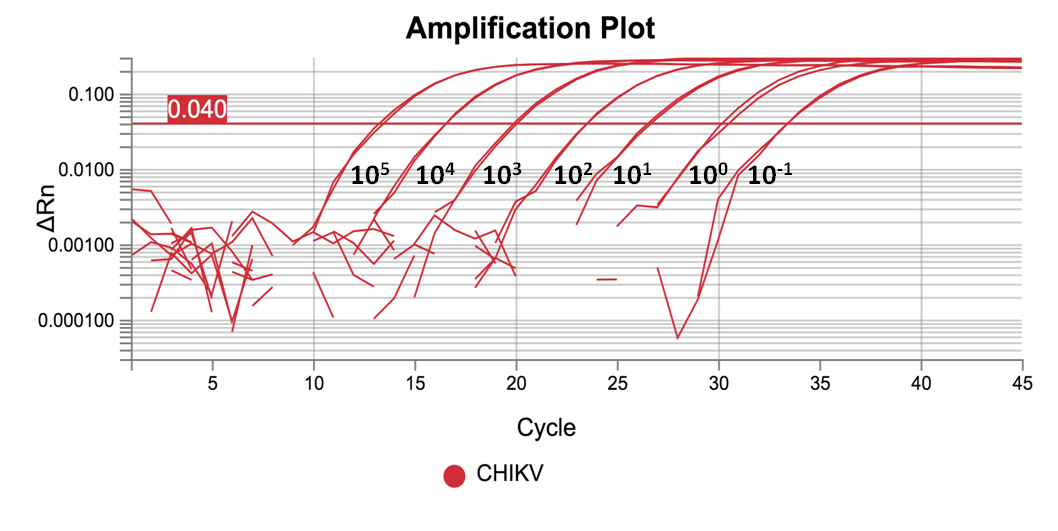
**

**Figure S6. Amplification plots obtained from the RT-qPCR during the analytical sensitivity analysis.**

**
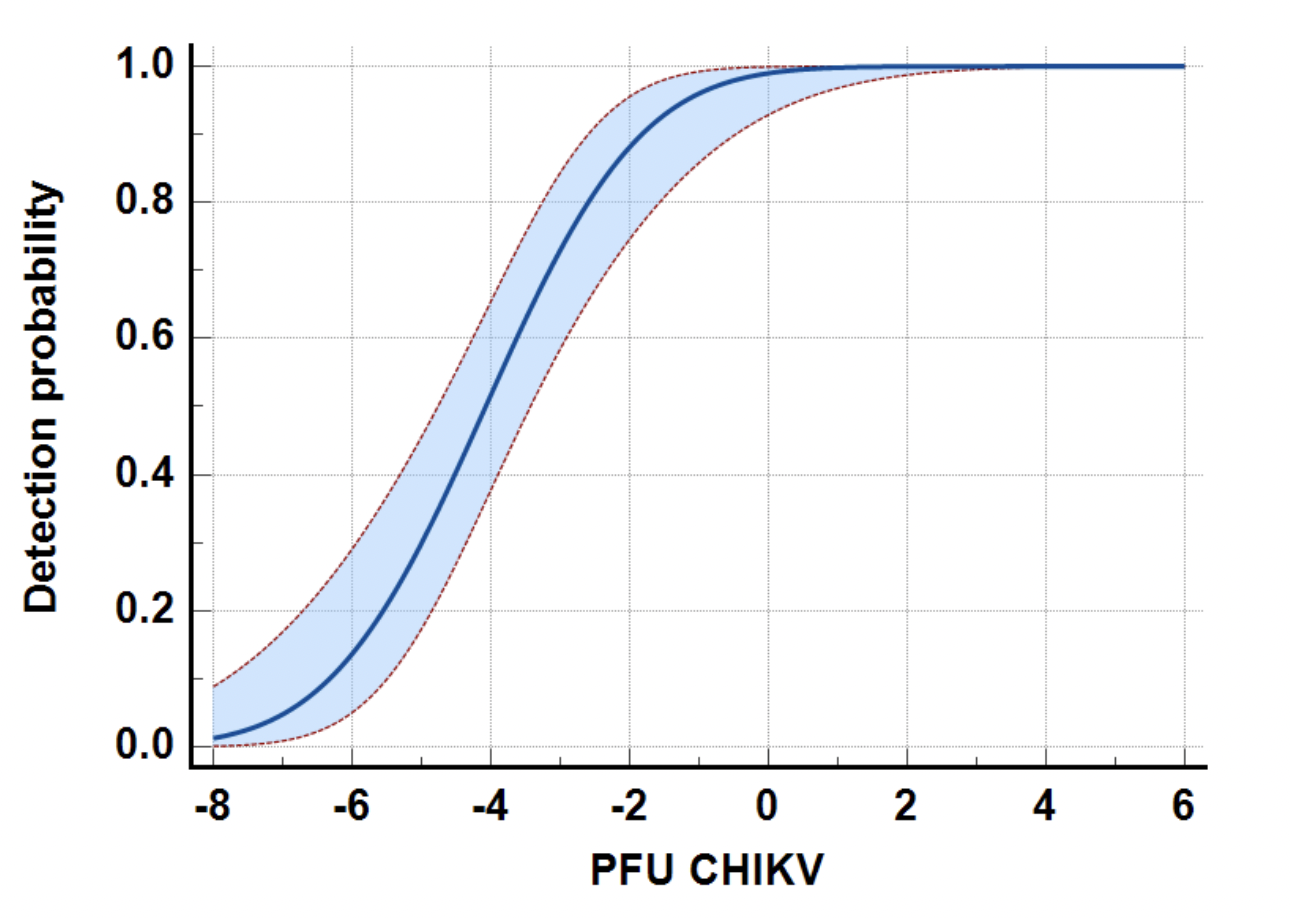
**

**Figure S7. Limit of detection of the RT-LAMP assay for CHIKV detection.** The probit regression analysis curve was generated from ten replicates of serial dilutions from human serum (10^5^ - 10^-7^ PFU) using MedCalc software. The limit of detection of RT-LAMP at 95% probability was −1.18 PFU of CHIKV with confidence interval from −2.08 to 0.45.

**
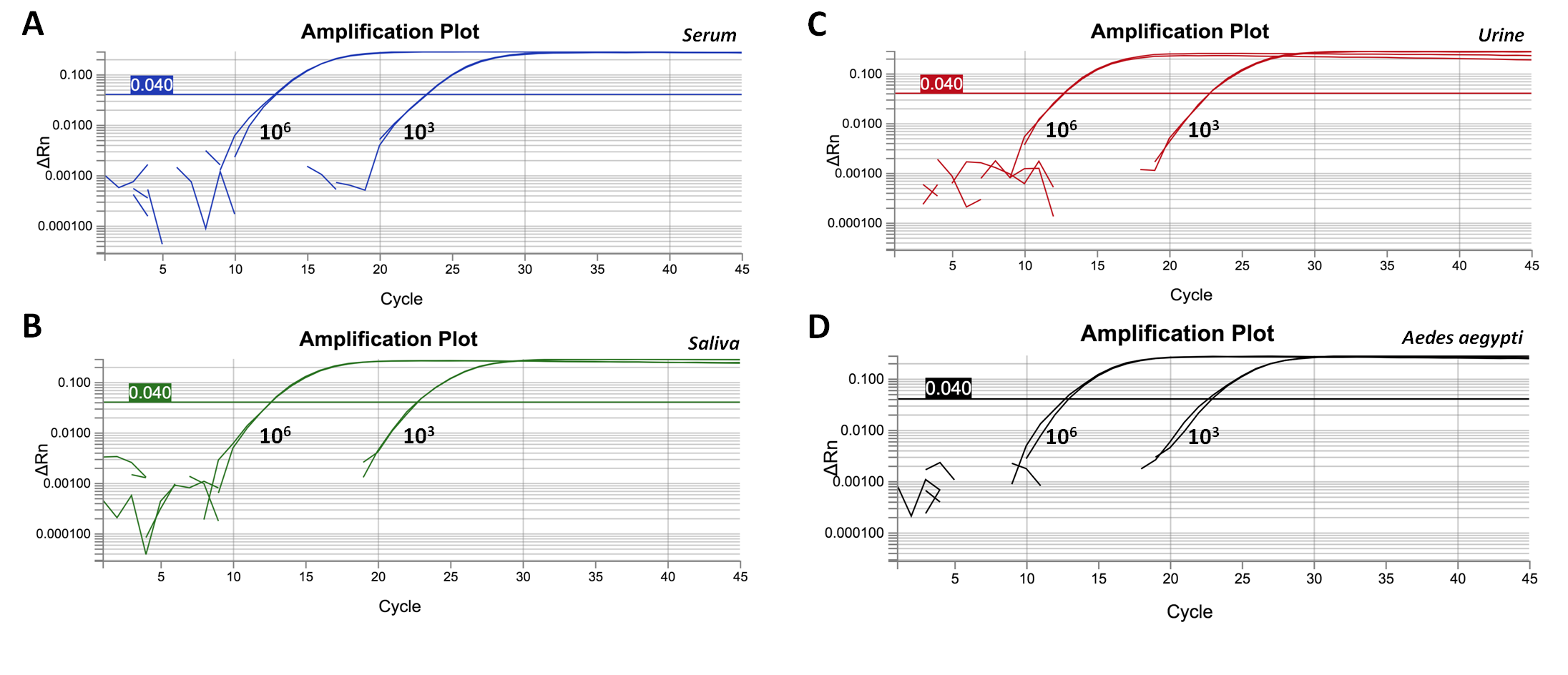
**

**Figure S8. Amplification plots obtained from the RT-qPCR in virus-spiked samples.** Human biological samples (serum, saliva, urine) and crude lysates from *Aedes aegypti* mosquitoes were spiked with high (10^6^ PFU/mL) and low (10^3^ PFU/mL) viral loads of CHIKV and then assayed by RT-qPCR assay.

**
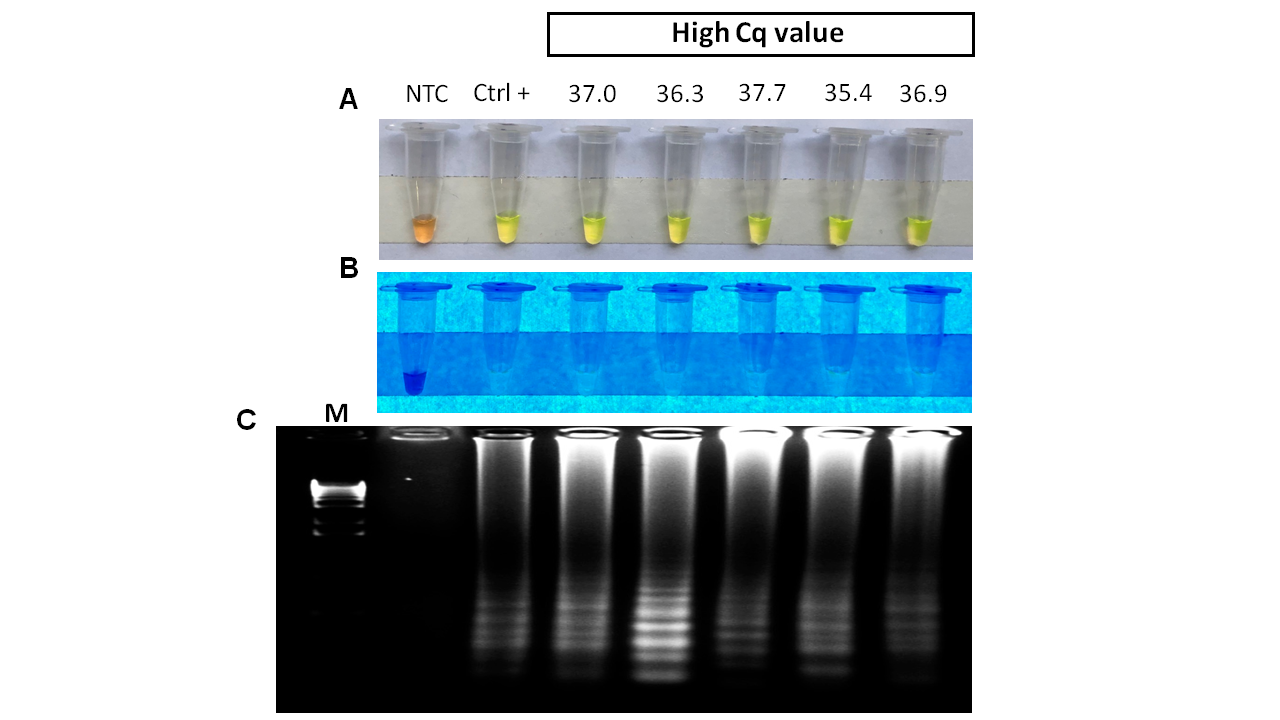
**

**Figure S9. Detection of CHIKV by RT-LAMP in patient samples at the RT-qPCR border line.** Serum samples at the detection threshold by RT-qPCR (Cq values ranging from 35.4 to 37.7) were assayed by RT-LAMP and all reactions showed a positive result. RT-LAMP products were visualized by three different methods: naked eye as visualized by addition of SYBR Green I (top panel), fluorescence under UV light (middle panel), or looking for a typical band pattern of a successful RT-LAMP reaction using gel electrophoresis (2 %) (bottom panel). M: molecular weight marker. Ctrl +: CHIKV culture supernatant (10^5^ PFU). NTC: non-template control.

**
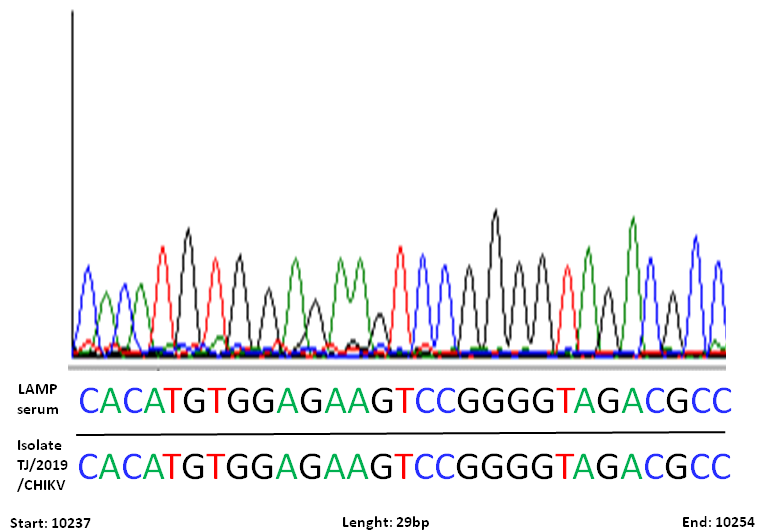
**

**Figure S10. Electropherogram of CHIKV RT-LAMP detected in human serum.** RT-LAMP amplicons from human serum were sequenced using Sanger method to confirm the identity of CHIKV. The region amplified was genome position 10237 to 10254. The obtained sequences were aligned against the CHIKV TJ/2019 reference isolate.

**
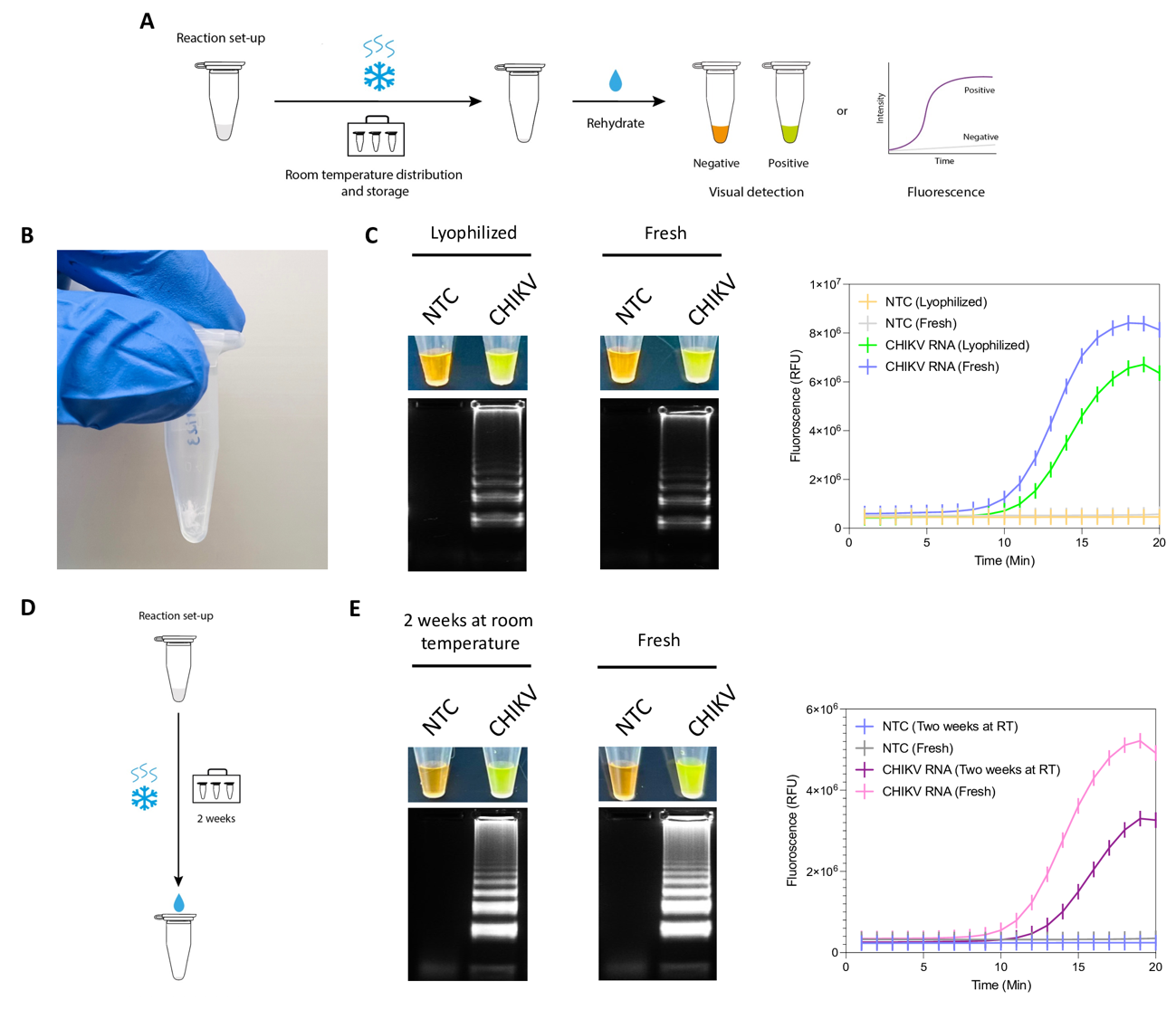
**

**Figure S11. Lyophilized RT-LAMP assay for CHIKV diagnostics.** Schematic showing the diagnostic workflow to develop lyophilized RT-LAMP reactions. RT-LAMP reactions were lyophilized and reactivated by adding water, indicating potential for room-temperature storage (A). Photograph illustrating an RT-LAMP reaction after lyophilization (B). Parallel evaluation (colorimetric and real-time detection) of lyophilized and fresh reactions using in vitro-transcribed RNA inputs (C). RT-LAMP reactions were lyophilized and kept at room temperature for two weeks and then reactivated by adding water (D). Syde-by-syde evaluation (colorimetric and real-time detection) of lyophilized reactions kept at room temperature for two weeks and fresh reactions using in vitro-transcribed RNA inputs (E). Legends: NTC: non-template control; RFU: relative fluorescence unit.

**Table S1.** Viruses used to evaluate the analytical specificity of the CHIKV RT-LAMP assay.

| **Viruses** | **Strain** | **GenBank access code** | **RT-LAMP data** |
| --- | --- | --- | --- |
| Flaviviridae, *Flavivirus*, Zika virus | PE243 | KX197192 | - |
| Flaviviridae, *Flavivirus*, Zika virus | MR766 | AY632535 | - |
| *Flaviviridae*, *Flavivirus*, Dengue virus serotype 1 | PE/97-42735 | EU259529 | - |
| *Flaviviridae*, *Flavivirus*, Dengue virus serotype 2 | PE/95-3808 | EU259569 | - |
| *Flaviviridae*, *Flavivirus*, Dengue virus serotype 3 | PE/02-95016 | KC425219 | - |
| *Flaviviridae, Flavivirus*, Dengue virus serotype 4 | PE/10-0081 | Unpublished | - |
| *Flaviviridae*, *Flavivirus*, Yellow fever virus | 17DD | DQ100292 | - |
| *Togaviridae*, *Alphavirus*, Mayaro virus | BR/Sinop/H307 | MH513597.1 | - |
| *Togaviridae*, *Alphavirus*, Chikungunya virus | PE2016-480 | Unpublished | + |

**Table S2.** LOD of the RT-LAMP assay for CHIKV detection in human serum.

| **CHIKV concentration (PFU)** | **Number of replicates** | **Number of positive results** | **Hit Rate in %** |
| --- | --- | --- | --- |
| 10^5^ | 10 | 10 | 100 |
| 10^4^ | 10 | 10 | 100 |
| 10^3^ | 10 | 10 | 100 |
| 10^2^ | 10 | 10 | 100 |
| 10^1^ | 10 | 10 | 100 |
| 10^0^ | 10 | 10 | 100 |
| 10^-1^ | 10 | 10 | 100 |
| 10^-2^ | 10 | 7 | 70 |
| 10^-3^ | 10 | 7 | 70 |
| 10^-4^ | 10 | 6 | 60 |
| 10^-5^ | 10 | 6 | 60 |
| 10^-6^ | 10 | 0 | 0 |
| 10^-7^ | 10 | 0 | 0 |
